# Impact of the COVID-19 pandemic on dengue in Brazil: Interrupted time series analysis of changes in surveillance and transmission

**DOI:** 10.1371/journal.pntd.0012726

**Published:** 2024-12-26

**Authors:** Kirstin Oliveira Roster, Tiago Martinelli, Colm Connaughton, Mauricio Santillana, Francisco A. Rodrigues

**Affiliations:** 1 Institute of Mathematics and Computer Science, University of São Paulo, São Carlos, SP, Brazil; 2 Mathematics Institute, University of Warwick, Coventry, United Kingdom; 3 London Mathematical Laboratory, London, United Kingdom; 4 Machine Intelligence Group for the Betterment of Health and the Environment, Network Science Institute, Northeastern University, Boston, Massachusetts, United States of America; 5 Center for Communicable Disease Dynamics, Harvard T.H. Chan School of Public Health, Boston, Massachusetts, United States of America; NIAID Integrated Research Facility, UNITED STATES OF AMERICA

## Abstract

Measures to curb the spread of SARS-CoV-2 impacted not only COVID-19 dynamics, but also other infectious diseases, such as dengue in Brazil. The COVID-19 pandemic disrupted not only transmission dynamics due to changes in mobility patterns, but also several aspects of surveillance, such as care seeking behavior and clinical capacity. However, we lack a clear understanding of the overall impact on dengue in different parts of Brazil and the contribution of individual causal drivers. In this study, we estimated the gap between expected and observed dengue cases in each Brazilian state from March to April 2020 using an interrupted time series design with forecasts from machine learning models. We then decomposed the gap into the contributions of pandemic-induced changes in disease surveillance and transmission dynamics, using proxies for care availability and care seeking behavior. Of 25 states in the analysis, 19 reported fewer dengue cases than predicted and the gap between expected and observed cases was largely explained by excess under-reporting, as illustrated by a reduction in observed cases below expected levels in early March 2020 in several states. A notable exception is the experience in the Southern states, which reported unusually large dengue outbreaks in 2020. These estimates of dengue case counts adjusted for under-reporting help mitigate some of the data gaps from 2020. Reliable estimates of changes in the disease burden are critical for anticipating future outbreaks.

## Author summary

The COVID-19 pandemic led to massive changes in human behavior and hospital capacity, which also affected the way other infectious diseases, like dengue, spread and our ability to track infections. Several countries observed unusual dengue outbreaks in 2020, including Brazil. However, we do not fully understand how the pandemic altered dengue case counts in different parts of the country and whether changes in human mobility or hospital capacity were more impactful. In this study, we examined how the COVID-19 pandemic impacted dengue in each Brazilian state in March and April 2020. We also tried to understand whether these changes were caused by insufficient hospital capacity to monitor dengue infections or actual changes in the number of people who were infected. We found that 19 out of 25 states reported fewer dengue cases than expected, mainly because patients were less likely to seek medical care and hospitals had lower capacity to treat and report illnesses. However, states in the South experienced unusually large dengue outbreaks in 2020, which cannot be explained by impacts of the COVID-19 pandemic. By adjusting for under-reporting, our estimates provide a clearer picture of the true dengue burden in 2020, which is important to prepare for future outbreaks.

## Introduction

Measures to curb the spread of SARS-CoV-2 impacted both the transmission and surveillance of other infectious diseases, such as dengue. During the COVID-19 pandemic, dengue infections in endemic regions in Asia and the Americas declined by an estimated 0.72 million cases [1, 2]. However, considerable variation was observed both between and within dengue endemic countries [1, 3] and the size of local effects of the pandemic on dengue remains unclear. Brazil reported high dengue incidence at national scale, especially early in the pandemic [1, 4, 5], though some states saw a reduction in cases upon onset of the pandemic [6]. Further, the mechanisms by which the pandemic altered the 2020 dengue season are not fully understood.

A possible mechanism is the change in dengue transmission patterns, driven by changes in human mobility, which by the current state of knowledge of the causes of dengue transmission could have had both positive and negative effects on dengue incidence. Given the short travel radius of *Aedes* mosquitoes, human mobility drives the geographic dispersal of the dengue virus [7–12], and some evidence suggests that mobility restrictions during the pandemic reduced dengue incidence, for example in the state of São Paulo [6]. Yet, changes in how people move are accompanied by changes in where people spend time. Spending less time in places with high mosquito density, such as construction sites, led to a reduction in dengue infections in Singapore, while workers who spent less time in air-conditioned offices experienced higher dengue incidence [3]. These conflicting findings suggest that the impact of the COVID-19 pandemic on dengue may be highly localized and context-dependent, and more sub-national analysis is needed.

Another possible mechanism is the change in surveillance systems. Under-reporting of dengue was a known challenge even before the pandemic, since as much as 84% of dengue infections are asymptomatic [13] and up to 95% of cases are not reported [14]. Ascertainment of a dengue infection relies on the (i) care-seeking behavior of the patient –which may depend on the trust in the health care system–, (ii) availability of care, and (iii) subsequent reporting upon diagnosis. All three of these factors may have been affected by the COVID-19 pandemic, as patients were less likely to seek care [15–17] and overburdened healthcare systems had a lower capacity for treatment and to fulfill reporting requirements [18]. At the national level, case fatality rates were within the typical range [4], though under-reporting has not yet been assessed at sub-regional scale, taking into account Brazil’s regional diversity in dengue endemicity and vector suitability [12, 19] as well as the robustness of local surveillance systems [15, 20]. Considerable variation has been observed in health care capacity and utilization both within and between states [21]. A typology of socioeconomic development and health care provision suggests that areas scoring highly on both socioeconomic development and health service provision are located primarily in the Southeast, especially in the state of São Paulo, while many areas in the North and Northeastern regions have lower socioeconomic development and health service provision. Given this variation in health care capacity, impacts on dengue reporting may also vary among states, again highlighting the need for localized analyses of the impacts of the COVID-19 pandemic [22].

In this study, we estimated the gap between expected and observed dengue cases in each Brazilian state using an interrupted time series analysis (ITSA) based on forecasts from machine learning models. We then decomposed this gap into the contribution of transmission dynamics and under-reporting, by estimating an adjusted dengue time series of cases that would have been observed in the absence of lower care availability and care seeking behavior during the COVID-19 pandemic.

The adjusted case counts help mitigate some of the data gaps from 2020. Reliable estimates of the true disease burden are necessary to anticipate not only the size of future outbreaks, but also the risk of secondary infections, which are more often associated with severe outcomes [23]. The sudden onset of the COVID-19 pandemic also represents an opportunity in the form of a natural experiment to study the role of different causal drivers of transmission, such as human mobility, which is critical to developing effective public health interventions.

## Materials and methods

### Data

**Fig 1:**
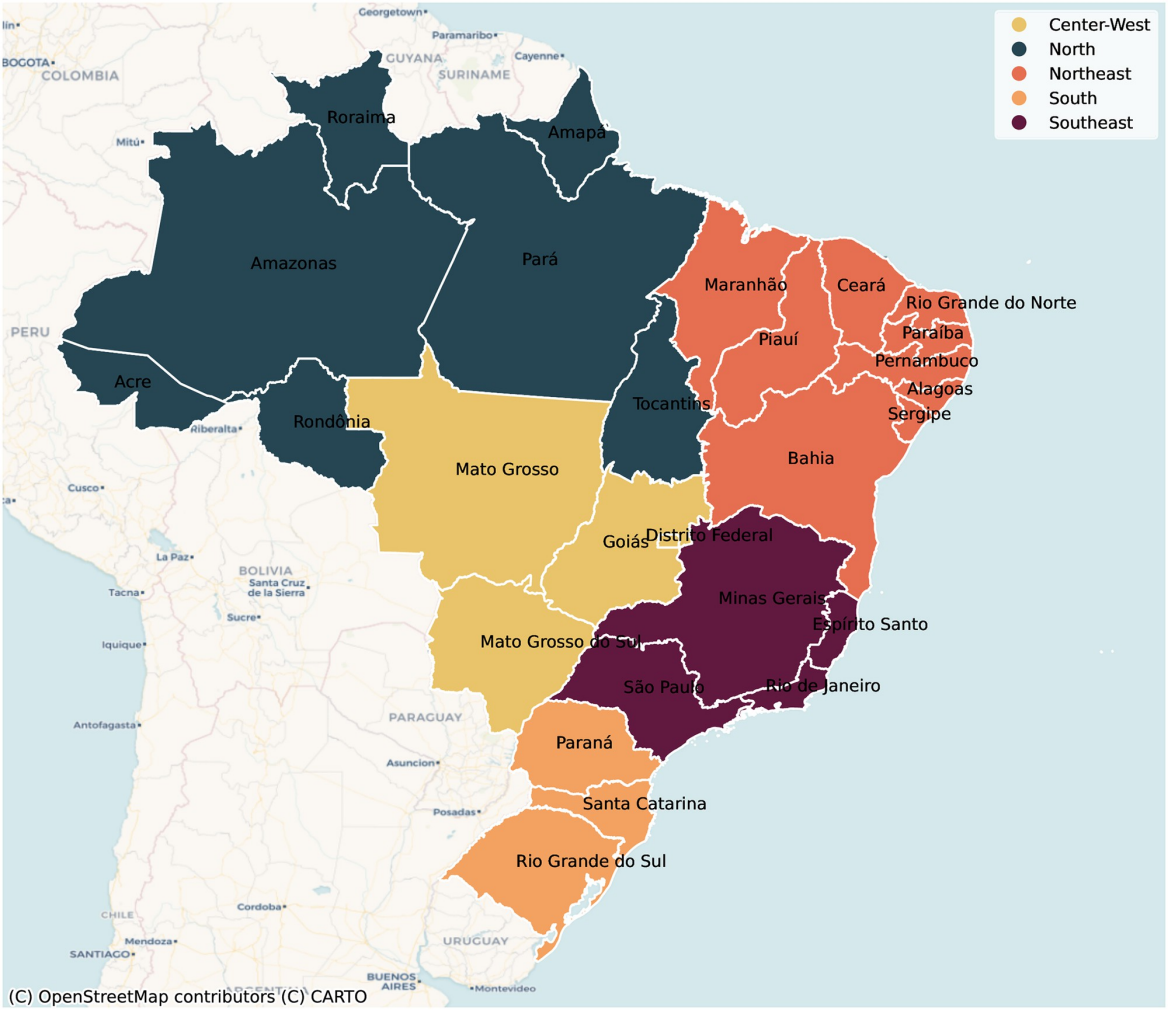
Study area. Map of Brazil with state boundaries, colored by region. Map data from OpenStreetMap, Copyright: Creative Commons, https://www.openstreetmap.org/copyright.

We extracted weekly observed dengue cases from 2014–2020 from the Brazilian Notifiable Diseases Information System (Sistema de Informação de Agravos de Notificação—SINAN) [24] for each of Brazil’s 27 Federative Units (26 states and the capital city, Distrito Federal) (Fig 1). Climate data were sourced from the Brazilian National Institute of Meteorology (Instituto Nacional de Meteorologia—INMET) over the same time period, specifically variables measuring rainfall, humidity, temperature (max, min, median) and wind speed [25]. To approximate changes in care-seeking behavior and care availability, we measured the number of treatments for conditions related to HIV infections (ICD-10 codes B20–24 and Z21) and elective hospitalizations from the Brazilian Ministry of Health (DATASUS) [26], respectively. We used Google Regional Mobility Reports to measure human mobility, specifically the change in time spent in transit stations during the pandemic, relative to a pre-pandemic baseline [27].

### Interrupted time series analysis

We developed an interrupted time series analysis (ITSA) [29, 30] to assess the causal impact of the COVID-19 pandemic on observed dengue cases. The interruption time was defined as the first week of March 2020, the start of the pandemic, when the largest mobility reductions were observed (S4 Fig). Accordingly, the data was split into a training set, consisting of all data up to the week of February 29th, 2020, and a forecast period, consisting of all data from the week of March 7th, 2020 until the wek of May 9th, 2020, resulting in a 10-week forecast horizon. Input features included 12 lags of reported dengue cases and all climate variables (rainfall; minimum, average, and maximum temperature; relative humidity; and average wind speed.

We performed four-fold expanding time series cross validation for model selection. The four validation sets each covered a full calendar year from 2016 to 2019, with training performed on all data preceding the validation year. For each validation fold, training and validation datasets were normalized using only information from the training sets, allowing for estimation of out-of-sample performance of the prediction models using mean absolute error (MAE).

During cross-validation, we compared several prediction algorithms, specifically random forest (number of trees: 150, maximum tree depth: 4, minimum number of samples per node: 3), XGBoost (maximum tree depth: 4, minimum number of samples per node: 3, learning rate: 0.05), gradient boosting regression (number of trees: 150, maximum tree depth: 4, minimum number of samples per node: 3, learning rate: 0.05), support vector machine regression (kernel: radial basis function, l2-regularization parameter: 10, tolerance *ϵ* = 0.1), a feed-forward neural network with single-step output (architecture: 3 layers with 128 units each, 128 x 128 x 128, activation function: rectified linear unit, optimizer: Adam, adaptive learning rate with starting value 0.001), a feed-forward neural network with multi-step output (architecture: 2 layers with 128 units each, 128 x 128, rectified linear unit activation, Adam optimization, and a learning rate of 0.00), a Bayesian structural time series model (using a Gamma prior distribution and previously described parametrization [31]), and a naïve model measuring the seasonal moving average, calculated as the historical average of weekly cases. We fitted separate models for each of the 10 forecast horizons, ranging from 1-week-ahead to 10-week-ahead predictions. From these models, we constructed 3 ensemble models, specifically (i) an average prediction of all models, (ii) an average prediction of the 3 best-performing models on the validation folds (random forest, XGBoost, gradient boosting regression), and (iii) a random forest meta-model that takes as inputs the predictions of all models. We constructed 95% prediction intervals of predictive performance using 200 bootstrapped samples of the predictions on the out-of-sample validation folds for each forecast horizon and state. A summary of the out-of-sample performance on the validation datasets is presented in S1 Table.

We selected the best-performing ensemble on the validation datasets—the average of the top 3 best models, which we trained on the full training set (2014–19) and used to make predictions on the forecast period (10 weeks from March 2020).

To measure the overall impact of the pandemic on dengue in 2020, we compared the predicted dengue cases from the ensemble model (i.e. those that we would have expected in the absence of the pandemic) with those that were actually observed. This expected-observed gap was calculated as the simple difference between observed and predicted cases. Two states, Amapá and Roraima, are excluded from the ITSA analysis, because of missing climate observations during part of 2020. We highlighted results for five sample states in the main text and presented full results in the supplement. The sample states were chosen to cover the five geographic regions of Brazil and to showcase a range of outcomes observed in our analyses.

### Reporting adjustment

We implemented a second ITSA to measure the extent to which lower-than-usual dengue cases could be explained by changes in surveillance, specifically by excess under-reporting, driven by reduced access to and availability of care during the pandemic. We leveraged two proxy time series to estimate under-reporting. First, changes in health care availability were approximated by data on elective hospitalizations. These elective procedures declined primarily due to supply-side factors, such as guidance to reserve hospital capacity for treatment of COVID-19 infections [32]. We expect capacity for treatment of mild cases of dengue to follow similar trends as capacity for elective hospitalizations. Second, reductions in care-seeking behavior were approximated by data on HIV-related treatments, which declined primarily due to impediments to accessing care early in the pandemic [15]. We expect behavioral factors reducing demand for dengue treatment to follow similar patterns.

For each of these proxy time series, we compared expected and observed quantities from March until April 2020 using an ensemble of predictions from a multi-step neural network and a Bayesian structural time series model, with architectures and hyperparameters defined as in the first ITSA. The percentage change between expected and observed HIV-related treatments and elective hospitalizations was then used to compute *adjusted dengue cases*, which may be interpreted as the dengue cases that would have been observed in the absence of changes in care seeking and care availability, respectively. Formally, we computed reporting-adjusted dengue cases d*, as $d_{t}^{*}=d_{t}\left(1-u_{t}\right)$, with the percentage change in reporting *u* determined by $u=\frac{a_{t}-\hat{a_{t}}}{a_{t}}$, where *d*_*t*_ are observed dengue cases at time *t*, *a*_*t*_ is the observed quantity of the reporting approximation, and $\hat{a_{t}}$ is the expected quantity of the reporting approximation predicted by ITSA. The under-reporting adjustment was performed only for states in which observed cases were lower than expected by the ITSA predictions.

All analyses were performed in Python version 3.9.13. We used the scikit-learn implementations for all machine learning models and the causalimpact implementation of the Bayesian structural time series (BSTS) model. All code is available at https://github.com/KRoster/impact-of-COVID-19-on-dengue.

## Results

**Fig 2:**
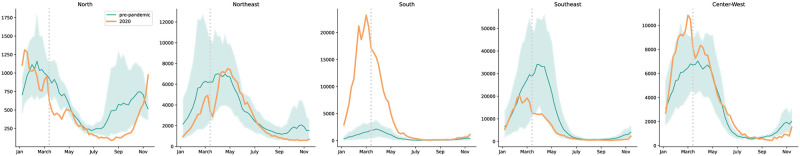
Observed dengue cases by epidemiological week and region. Average and 95% confidence interval of weekly dengue cases from 2014 to 2019 (teal) and weekly observed dengue cases in 2020 (orange). The start of the COVID-19 pandemic is indicated by vertical gray dotted lines.

At regional level, dengue cases were comparable to prior years in the North and Northeast, atypically high in the South and Center-West, and comparable except for a dip in cases in March and April in the Southeast (Fig 2). Of 25 states in the ITSA, 19 reported fewer dengue cases than predicted (Table 1), though observed cases were within the 95% prediction interval for most states (S2 Fig). The remaining 6 states reported more cases than predicted (Table 1), with observed cases falling outside the 95% prediction interval and thus suggesting unusually large dengue outbreaks (S2 Fig).

**Table 1:** Total observed and expected dengue cases from March to April 2020, by state and region.

Region	State	Observed cases	Prediction (ensemble)	Gap (expected—observed)	Gap (percentage of expected)
Center-west	Distrito Federal	19,255	18,193	-1,062	-6%
	Goiás	20,863	21,687	824	4%
	Mato Grosso	10,355	13,113	2,758	21%
	Mato Grosso do Sul	18,052	25,401	7,349	29%
North	Acre	574	1,097	523	48%
	Amazonas	1,185	1,317	132	10%
	Pará	959	1,196	237	20%
	Rondônia	1,302	1,094	-208	-19%
	Tocantins	574	613	39	6%
Northeast	Alagoas	390	764	374	49%
	Bahia	25,440	15,645	-9,795	-63%
	Ceará	7,138	9,768	2,630	27%
	Maranhão	753	1,699	946	56%
	Paraíba	958	2,403	1,445	60%
	Pernambuco	2,823	3,439	616	18%
	Piauí	598	956	358	37%
	Rio Grande do Norte	1,231	1,387	156	11%
	Sergipe	104	297	193	65%
South	Paraná	120,324	140,230	19,906	14%
	Rio Grande do Sul	2,548	1,658	-890	-54%
	Santa Catarina	5,112	1,256	-3,856	-307%
Southeast	Espírito Santo	1,985	4,686	2,701	58%
	Minas Gerais	32,867	32,633	-234	-1%
	Rio de Janeiro	1,220	3,647	2,427	67%
	São Paulo	72,449	109,860	37,411	34%

### Southeast

**Fig 3:**
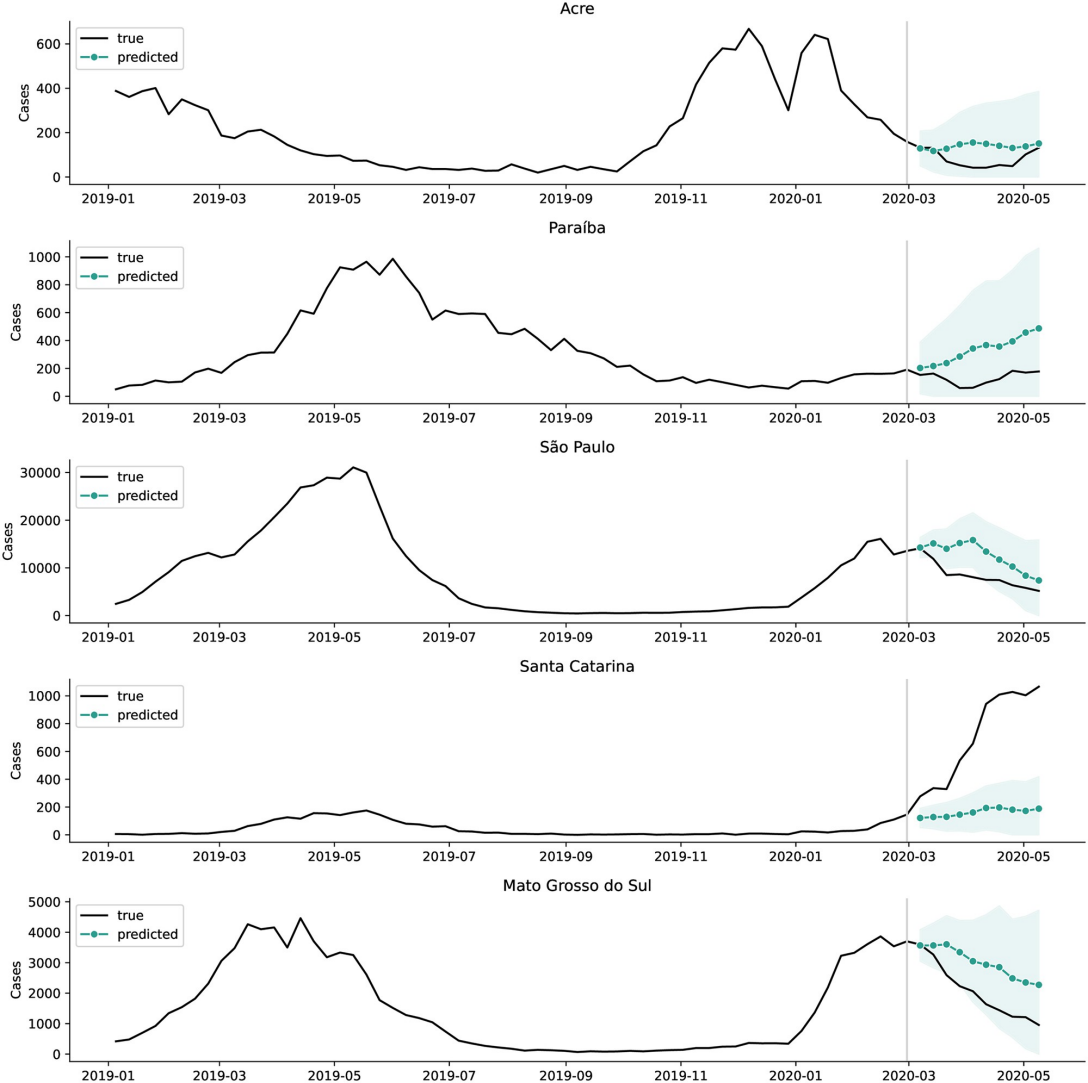
Observed and expected dengue cases in five sample states. Observed dengue cases (black) and 1- to 10-week forecasts (teal) with 95% prediction intervals (shaded area).

**Fig 4:**
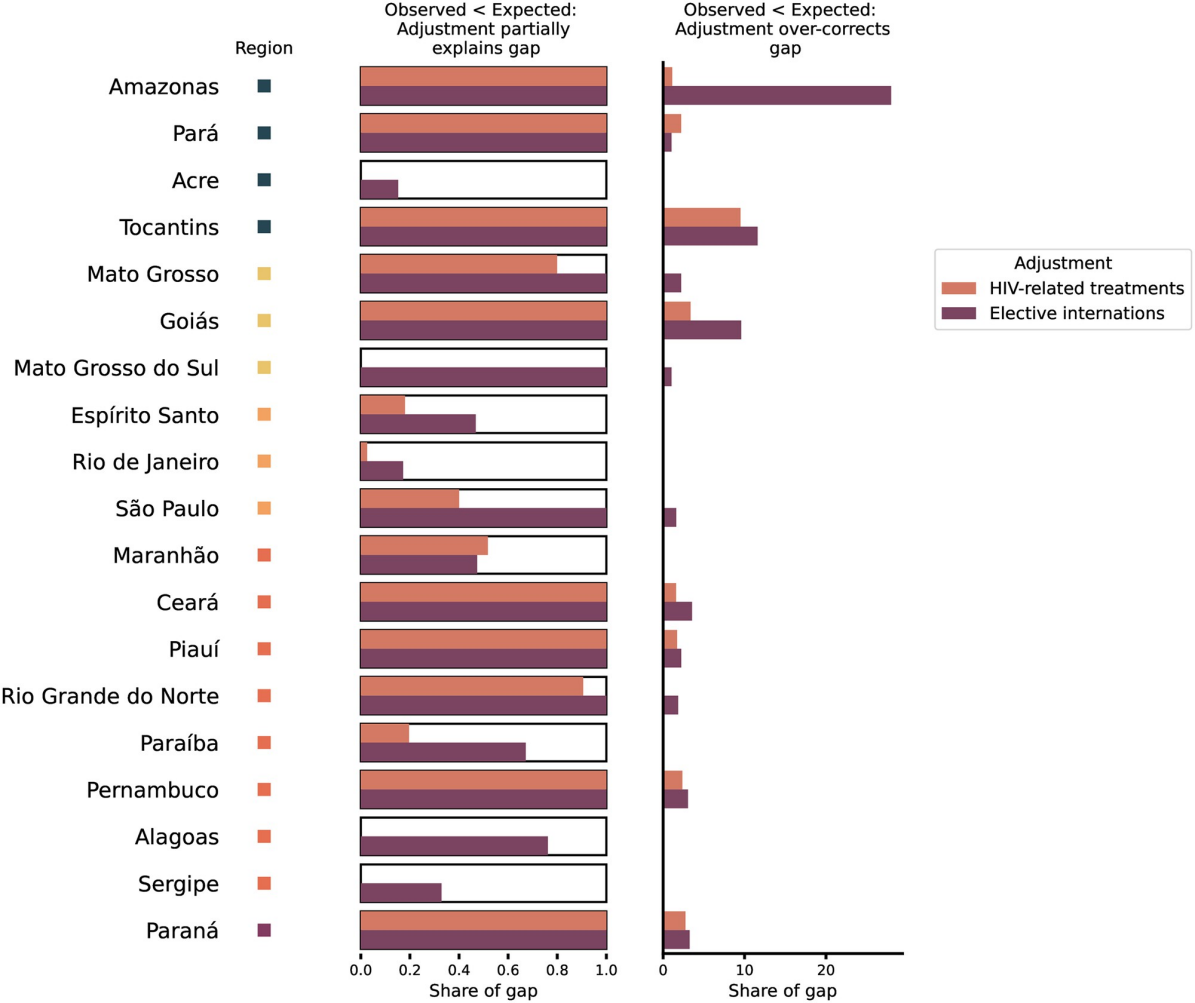
Proportion of the observed-expected gap explained by under-reporting. Total gap between expected and observed cases in March and April 2020 (black boxes) and the proportion of the gap that is explained by under-reporting adjustments using HIV-related treatments (orange) and elective hospitalizations (purple) proxies. Columns show (i) states where observed cases are lower than expected cases, and at least one of the two adjustments is within the observed-expected gap (Observed < adjusted < expected) (left column) and (ii) states where the adjustment over-corrects for the gap, such that the adjustment is greater than the prediction (Observed < expected < adjusted) (right column). All x-values are expressed as shares of the observed-expected gap.

**Fig 5:**
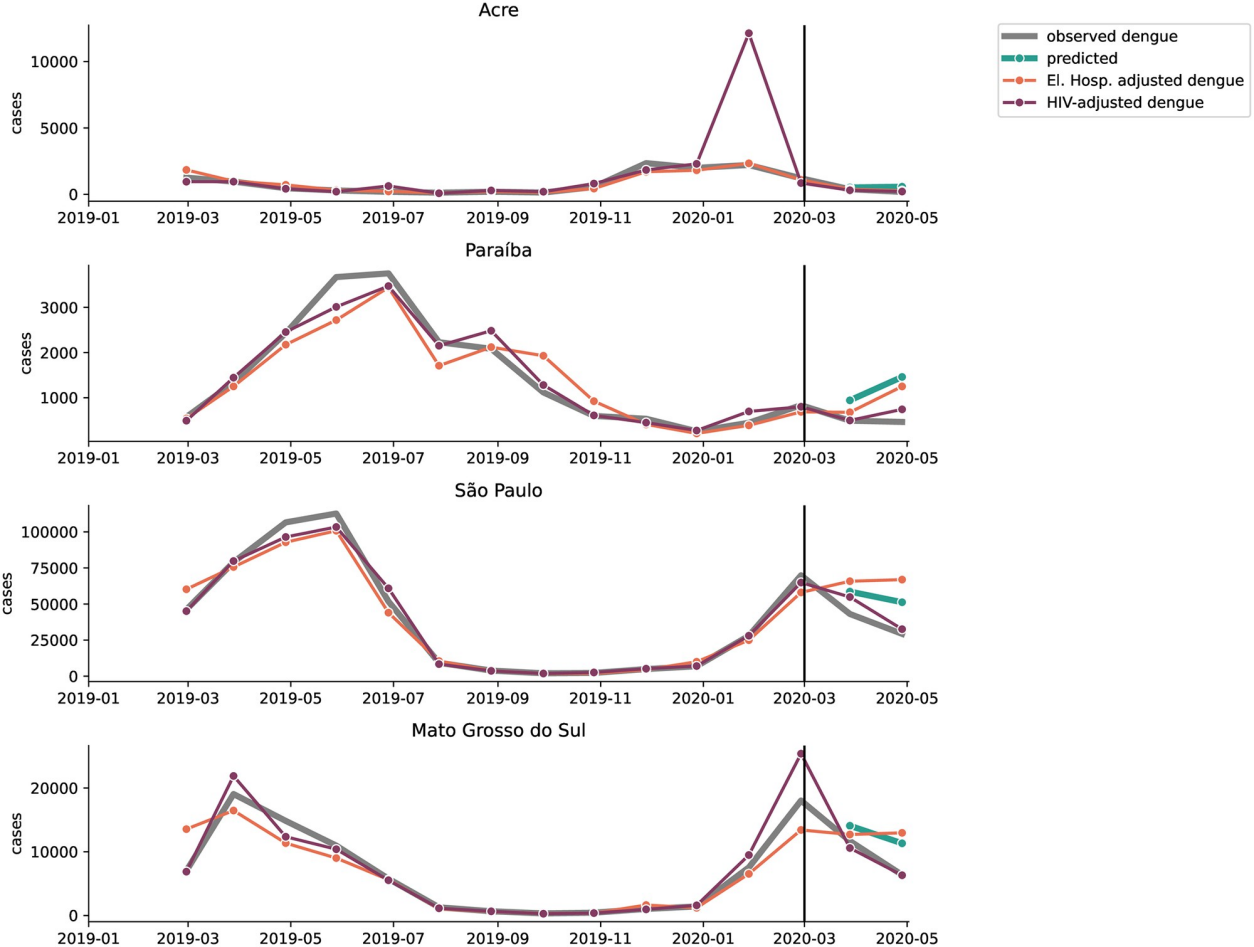
Observed, expected, and under-reporting-adjusted dengue cases over time in sample states. Observed dengue cases (dark gray), predicted dengue cases (teal), and under-reporting-adjusted cases using HIV-related treatments (orange) and elective hospitalizations proxies (purple).

States in the Southeast observed fewer dengue infections than expected (Table 1), except for Minas Gerais, where observed cases differed from expected cases by less than 1%. In São Paulo, the season started with higher-than-typical incidence in the first half of February (Fig 2 and S1 Fig), but cases began to decline in epidemiological week 8 (February 22nd), resulting in a cumulative gap of 37 thousand fewer cases (34%) than expected in March and April of 2020 (Table 1 and Fig 3). Adjusting for reduced care availability in São Paulo resulted in a case count that closely followed the predicted time series, while reduced care seeking accounted for only 40% of the expected-observed gap (Figs 4 and 5). In Rio de Janeiro, the gap of 3.6 thousand cases (67%) was only partially explained by under-reporting, suggesting that other factors are needed to explain the low observed case count (Fig 4).

The government of Espírito Santo stopped using the online disease notification system in 2020 [24], leading to clearly evidenced and sudden under-reporting (S1 Fig), which helped validate our ITSA results. The models identified this break in the data generation mechanism and predicted higher cases than reported (S2 Fig).

### South

All three states in the South reported much larger dengue outbreaks in 2020 than in prior years (Fig 2). Santa Catarina observed 3,856 (307%) more cases than expected and Rio Grande do Sul observed 890 (54%) more than expected (Table 1 and Fig 3). Paraná also reported atypically high dengue cases (Fig 2), though the excess cases occurred largely before the start of the pandemic, peaking in the week of March 14th (S1 and S2 Figs).

### Center-West

Dengue cases in three of four federative regions in the Center-West were higher than observed in previous years (in Mato Grosso, Distrito Federal, Mato Grosso do Sul, see S1 Fig). However, cases were still lower than predicted from the ITSA in Mato Grosso do Sul (29%) and in Mato Grosso (21%) (Table 1 and S2 Fig). Observed cases were roughly as expected in Goiás (4% more than expected) and Distrito Federal (6% fewer than expected) in spite of a notable dip in cases around the onset of the pandemic (Table 1 and S2 Fig). Under-reporting either fully explained (e.g. adjustment for care access in Mato Grosso) or over-corrected for the observed-expected gap, especially in Goiás (Fig 4 and S3 Fig).

### Northeast

Eight of the nine states in the Northeast experienced fewer cases than predicted (Table 1) and than had been seen in prior years (S1 Fig). In half of these states, under-reporting fully explained and over-corrected for the expected-observed gap (Ceará, Piauí, Rio Grande do Norte, and Pernambuco), while under-reporting only partially explained the gap in the other half of the states (Maranhão, Paraíba, Alagoas, Sergipe) (Fig 3). Ceará, for example, began the season with typical incidence, followed by a dip in cases in mid-March, and remained below typical incidence for the rest of the dengue season (S1 Fig). The gap between expected and observed cases in March and April amounted to 2,630 cases (27%) (Table 1), which was fully explained by both reporting adjustments (Fig 4). Bahia deviated from the other states in the Northeast, with its higher-than-expected incidence (Table 1). However, the outbreak size in Bahia was not as unprecedented as in the South, since 2020 case counts were almost as large as during the 2016 outbreak and the outbreak timing was comparable to the 2019 season.

### North

The Northern region experiences more variation in the timing of dengue cases than other parts of Brazil and outbreaks tend to be less intensive and less seasonal, making prediction of dengue cases in this region more challenging [12, 19]. Relative to other regions, outbreaks in the Northern states tend to peak earlier (December-February) [19] (S1 Fig) and are thus less likely to have been impacted by the pandemic. Observed time series were comparable to previous years in all states except for the state of Amazonas, which observed higher case counts than usual in January (S1 Fig). Observed cases in all states were both higher and lower than expected at some time points in the predictions (S2 Fig), with total observed cases lower than expected in all states except for Rondônia (Table 1). The size of the gap ranged from 6% in Tocantins to 48% in Acre (Table 1). Acre was the only state in the North where under-reporting adjustment did not explain the expected-observed gap (Fig 3).

## Discussion

In an interrupted time series analysis of dengue cases during the COVID-19 pandemic, most states in Brazil, with exception of the South, reported fewer dengue cases than expected. Observed cases fell within the 95% prediction intervals for each of these states—allowing for the possibility of no pandemic impact; yet other dengue endemic countries in Latin America and Asia also reported a decline in dengue cases during the pandemic [1], and several states reported a pronounced dip in infections at the start of the pandemic in early March 2020, thus strengthening the finding that much of Brazil reported fewer dengue cases than would have been expected in the absence of the COVID-19 pandemic. Model adjustments for under-reporting due to changes in care seeking behavior and care availability fully explained the gap between expected and observed cases in many states.

A notable exception is the experience in the Southern states, which reported unusually large outbreaks in 2020. The South of Brazil has historically had lower dengue incidence due to the region’s more temperate climate, though climatic changes are creating more favorable conditions for mosquitoes, which together with low levels of population immunity have increased the risk of dengue transmission [34, 35].

While reports of treatment delays, reduced capacity, and drops in care-seeking during the pandemic are abundant [15–18, 36], it has been difficult to quantify the impact of under-reporting on infectious diseases and separate their impact from changes in transmission. Nationally, case fatality rates (CFR) were within the typical range in 2020 [1], which has been taken as evidence that no widescale under-reporting took place in 2020. However, the CFR increased relative to 2019 [37] and averaging over the entire country and dengue season may hide regionally and temporally localized under-reporting. Increased numbers of first exposures in the South with lower mortality risk could further skew the CFR in that region.

This study has several limitations. Firstly, our causal effect estimates rely on the quality of our predictive models [29] and dengue forecasting is an inherently difficult task [38]. To mitigate some of the risks, we compared eight different machine learning algorithms and three ensemble approaches, which produced qualitatively similar results. We further highlighted the limits of our predictions using confidence intervals and out-of-sample performance estimates on pre-pandemic data (S1 Table).

Secondly, in the absence of data on excess under-reporting during the pandemic, we relied on proxy time series to estimate changes in care availability and demand. These approximations may be confounded by other changes during the pandemic, such as mobility reductions. However, mobility reductions in Brazil occurred synchronously across the country, even though policy interventions like school closures, were implemented at different time points (S4 Fig). Mobility reduced sharply in the first two weeks of March 2020, and did not rebound to pre-pandemic levels until the end of the year. Any changes in transmission caused by mobility should take a similar shape. In many states, such as São Paulo, Piauí, and Ceará, under-reporting estimates showed a short sharp drop in dengue cases in March 2020, with a quick rebound, consistent with sudden changes in capacity as the health care system prepared for the first COVD-19 wave. In the state of São Paulo, for example, the reduction in dengue cases tracks very closely with the reduction in elective hospitalizations, a proxy for care availability. This suggests that the reduction in reported cases was affected by changes in capacity to treat dengue infections, not only by altered transmission patterns due to mobility reductions [6].

Finally, we focused on separating the impacts of changes in both reporting and transmission of dengue. However, dengue transmission is influenced by a complex interplay of different factors, several of which may have also been impacted by the COVID-19 pandemic, and which could bias the results of our under-reporting analysis. Some evidence already exists on the negative impacts on dengue transmission of reduced mobility [6], school closures [1], and reductions in time spent in non-residential areas [1]. Other factors that may have altered dengue incidence in 2020 include changes in vector abundance due to climate conditions, reduced vector control activities, or insecticide resistance and policies to reduce the spread of SARS-CoV-2 which may have changed human-mosquito interactions, such as the closure of public parks and recreational areas. More research is needed to untangle the different drivers of dengue transmission across Brazil, especially the role of changes in human susceptibility, increased time spent at home during the pandemic, and changes in vector control and dengue education programs.

We provided state-level estimates of the impact of the COVID-19 pandemic on dengue incidence and measured the extent to which changes in reporting relative to changes in transmission explained reductions in observed dengue cases. Closing the data gap on dengue infections in 2020 is important to understand population immunity and severe dengue risk from secondary infections as well as to track changes in dengue dynamics over time.

## Supporting information

S1 Table Out-of-sample model performance on validation datasets. (DOCX)

S1 Fig Observed dengue cases by epidemiological week and state. Average and 95% confidence interval of weekly dengue cases from 2014 to 2019 (teal) and weekly observed dengue cases in 2020 (orange).
(DOCX)

S2 Fig Expected and observed dengue cases, by state. Observed dengue cases (black) and 1- to 10-week forecasts (teal) with 95% confidence intervals (shaded area).
(DOCX)

S3 Fig Monthly observed and under-reporting-adjusted dengue cases, by state. (DOCX)

S4 Fig Change in transit station mobility, relative to pre-pandemic baseline, by region, and dengue and COVID-19 cases. Mean and 95% confidence interval of Google mobility index (gray) across cities in each region. Orange vertical dotted lines highlight epidemiological weeks 11-13. B) COVID-19 cases (teal) and dengue cases in 2020 (dark orange) and from 2014-2019 (light orange), with shaded areas representing the 95% confidence interval over the six-year period.
(DOCX)
