# Correction: Impact of the COVID-19 pandemic on dengue in Brazil: Interrupted time series analysis of changes in surveillance and transmission

**DOI:** 10.1371/journal.pntd.0013030

**Published:** 2025-04-17

**Authors:** Kirstin Oliveira Roster, Tiago Martinelli, Colm Connaughton, Mauricio Santillana, Francisco A. Rodrigues

The following information is missing from the funding statement: This study was financed in part by the Coordenação de Aperfeiçoamento de Pessoal de Nível Superior – Brasil (CAPES) – Finance Code 001.

The correct funding statement is as follows: K.O.R. gratefully acknowledges the support of the São Paulo Research Foundation (FAPESP) grant number 2019/26595-7. F.A.R. acknowledges CNPq (grant 309266/2019-0) and FAPESP (grant 19/23293-0) for the financial support given for this research. This study was financed in part by the Coordenação de Aperfeiçoamento de Pessoal de Nível Superior – Brasil (CAPES) – Finance Code 001. M.S. acknowledges funding from the National Institute of General Medical Sciences of the National Institutes of Health under award number R01GM130668. The content is solely the responsibility of the authors and does not necessarily represent the official views of the National Institutes of Health. The funders had no role in study design, data collection and analysis, decision to publish, or preparation of the manuscript.
