# Supplementary material for: Impact of the COVID-19 pandemic on dengue in Brazil: Interrupted time series analysis of changes in surveillance and transmission
Source: PLoS Negl Trop Dis. 2024 Dec 26;18(12):e0012726. doi: 10.1371/journal.pntd.0012726 (PMC11709241; doi:10.1371/journal.pntd.0012726)
Supplement: S1 Table — (DOCX) [file pntd.0012726.s001.docx]

**S1 Table. Out-of-sample model performance on validation datasets, using an ensemble of the top 3 best machine learning models.**

| Forecast horizon (weeks ahead) | Mean absolute error (MAE) of predictions | Average observed dengue cases over time period |
| --- | --- | --- |
| 1 | 177.17 | 651.31 |
| 2 | 225.82 | 649.08 |
| 3 | 274.27 | 646.90 |
| 4 | 312.11 | 644.88 |
| 5 | 349.62 | 642.96 |
| 6 | 390.61 | 641.24 |
| 7 | 427.77 | 639.68 |
| 8 | 466.82 | 638.06 |
