# Supplementary material for: Impact of the COVID-19 pandemic on dengue in Brazil: Interrupted time series analysis of changes in surveillance and transmission
Source: PLoS Negl Trop Dis. 2024 Dec 26;18(12):e0012726. doi: 10.1371/journal.pntd.0012726 (PMC11709241; doi:10.1371/journal.pntd.0012726)
Supplement: S1 Fig — Average and 95% confidence interval of weekly dengue cases from 2014 to 2019 (teal) and weekly observed dengue cases in 2020 (orange). (DOCX) [file pntd.0012726.s002.docx]

**S1 Fig. Observed dengue cases by epidemiological week and state.** Average and 95% confidence interval of weekly dengue cases from 2014 to 2019 (teal) and weekly observed dengue cases in 2020 (orange).
