# Supplementary material for: Impact of the COVID-19 pandemic on dengue in Brazil: Interrupted time series analysis of changes in surveillance and transmission
Source: PLoS Negl Trop Dis. 2024 Dec 26;18(12):e0012726. doi: 10.1371/journal.pntd.0012726 (PMC11709241; doi:10.1371/journal.pntd.0012726)
Supplement: S2 Fig — Observed dengue cases (black) and 1- to 10-week forecasts (teal) with 95% confidence intervals (shaded area). (DOCX) [file pntd.0012726.s003.docx]

**S2 Fig. Expected and observed dengue cases, by state.** Observed dengue cases (black) and 1- to 10-week forecasts (teal) with 95% confidence intervals (shaded area).
