# Supplementary material for: Impact of the COVID-19 pandemic on dengue in Brazil: Interrupted time series analysis of changes in surveillance and transmission
Source: PLoS Negl Trop Dis. 2024 Dec 26;18(12):e0012726. doi: 10.1371/journal.pntd.0012726 (PMC11709241; doi:10.1371/journal.pntd.0012726)
Supplement: S3 Fig — (DOCX) [file pntd.0012726.s004.docx]

**S3 Fig. Monthly observed and under-reporting-adjusted dengue cases, by state.**
