# Supplementary material for: Impact of the COVID-19 pandemic on dengue in Brazil: Interrupted time series analysis of changes in surveillance and transmission
Source: PLoS Negl Trop Dis. 2024 Dec 26;18(12):e0012726. doi: 10.1371/journal.pntd.0012726 (PMC11709241; doi:10.1371/journal.pntd.0012726)
Supplement: S4 Fig — Mean and 95% confidence interval of Google mobility index (gray) across cities in each region. Orange vertical dotted lines highlight epidemiological weeks 11-13. B) COVID-19 cases (teal) and dengue cases in 2020 (dark orange) and from 2014-2019 (light orange), with shaded areas representing the 95% confidence interval over the six-year period. (DOCX) [file pntd.0012726.s005.docx]

**S4 Fig. Change in transit station mobility, relative to pre-pandemic baseline, by region, and dengue and COVID-19 cases.** A) Mean and 95% confidence interval of Google mobility index (gray) across cities in each region. Orange vertical dotted lines highlight epidemiological weeks 11-13. B) COVID-19 cases (teal) and dengue cases in 2020 (dark orange) and from 2014-2019 (light orange), with shaded areas representing the 95% confidence interval over the six-year period.

A)


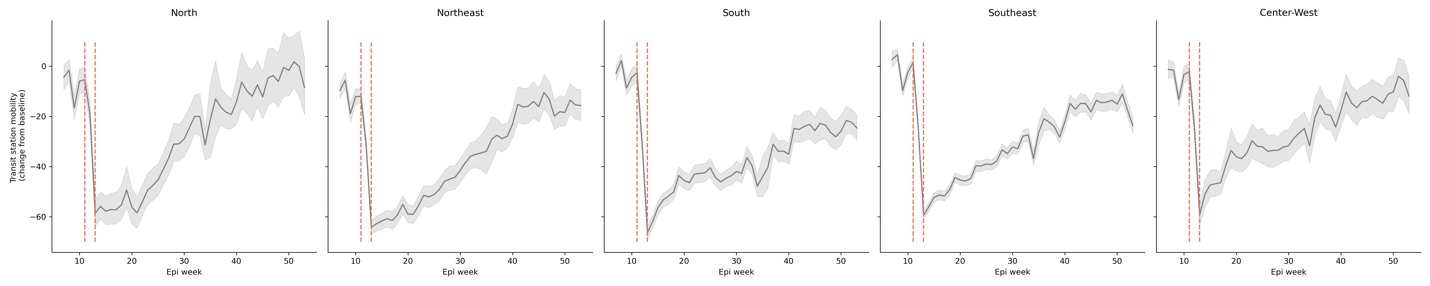


B)
